# Unraveling the Optimum Latent Structure of Attention-Deficit/Hyperactivity Disorder: Evidence Supporting ICD and HiTOP Frameworks

**DOI:** 10.3389/fpsyt.2021.666326

**Published:** 2021-05-14

**Authors:** Rapson Gomez, Lu Liu, Robert Krueger, Vasileios Stavropoulos, Jenny Downs, David Preece, Stephen Houghton, Wai Chen

**Affiliations:** ^1^School of Science, Psychology, and Sport, Federation University, Ballarat, VIC, Australia; ^2^Peking University Sixth Hospital/Institute of Mental Health, Beijing, China; ^3^National Clinical Research Center for Mental Disorders (Peking University Sixth Hospital) & NHC Key Laboratory of Mental Health (Peking University), Beijing, China; ^4^Department of Psychology, University of Minnesota, Minneapolis, MN, United States; ^5^Department of Psychology, Victoria University, Melbourne, VIC, Australia; ^6^Telethon Kids Institute, Nedlands, WA, Australia; ^7^School of Physiotherapy and Exercise Science, Curtin University, Perth, WA, Australia; ^8^School of Psychology, Curtin University, Perth, WA, Australia; ^9^Graduate School of Education, University of Western Australia, Perth, WA, Australia; ^10^Mental Health Service, Fiona Stanley Hospital, Perth, WA, Australia; ^11^School of Medicine, University of Notre Dame Australia, Fremantle, WA, Australia; ^12^Department of Psychology, Murdoch University, Perth, WA, Australia

**Keywords:** children, ADHD, CFA models, ESEM model, S-1 bi-factor CFA models, DSM- 4, ICD-10, HiTOP

## Abstract

Attention Deficit/hyperactivity disorder (ADHD) is conceptualized differently in the Diagnostic and Statistical Manual (DSM-5), the International Classification of Diseases-10 (ICD-10), and the Hierarchical Taxonomy of Psychopathology (HiTOP) frameworks. This study applied independent cluster confirmatory factor analysis (ICM-CFA), exploratory structure equation model with target rotation (ESEM), and the S-1 bi-factor CFA approaches to evaluate seven ADHD models yielded by different combinations of these taxonomic frameworks. Parents and teachers of a community sample of children (between 6 and 12 years of age) completed the Disruptive Behavior Rating Scale (for ADHD symptoms) and the Strengths and Difficulties Questionnaire (for validation). Our findings for both parent and teacher ratings provided the most support for the S-1 bi-factor CFA model comprised of (i) a g-factor based on ICD-10 impulsivity symptoms as the reference indicators and (ii) inattention and hyperactivity as specific factors. However, the hyperactivity-specific factor lacked clarity and reliability. Thus, our findings indicate that ADHD is best viewed as a disorder primarily reflecting impulsivity, though with a separable inattention (but no hyperactivity) component, i.e., “ADID (attention deficit/impulsivity disorder).” This model aligns with the HiTOP proposals.

## Introduction

For Attention Deficit/Hyperactivity Disorder (ADHD), the latest fifth edition of the Diagnostic and Statistical Manual [DSM-5; (1)] has retained the same comparable nine inattention (IA), six hyperactivity (HY), and three impulsivity (IM) symptoms as in previous editions [DSM-IV; (2, 3)]. As in previous editions, the HY and IM symptoms are conceptualized as a single dimension (HY/IM). In the International Classification of Diseases-10 [ICD-10; (4)], ADHD is referred to as Hyperkinetic Disorder (HD). Although DSM-5 and ICD-10 have the same sets of symptoms for ADHD/HD, they are grouped differently. Unlike DSM-5, the HY and IM symptoms in ICD-10 are considered as distinct groups. Additionally, the “talkative” symptom (classified as a HY symptom in the DSM-5) is designated as an IM symptom in ICD-10; these HY and IM symptom groups in ICD-10 have been referred to as “motoric HY/IM” and “verbal HY/IM,” respectively (5).

Recently, a dimensional model of psychopathology called the Hierarchical Taxonomy of Psychopathology [HiTOP; (6, 7) has been proposed. HiTOP is a data-driven hierarchical, dimensional classification system (HiTOP) that continues to be refined. In the current version of HiTOP, several broad dimensional super spectra (for example, internalizing and externalizing) are at the highest level. Below this are six spectra (somatoform, internalizing, thought disorder, disinhibited externalizing, antagonistic externalizing, and detachment). At the level below the spectra are subfactors, and below this are the syndromes and disorders. These syndromes and disorders do not correspond to the broad disorder composites (for instance ADHD) listed in the more traditional classification systems (for instance, DSM-5 and ICD-10) but represent specific dimensions (such as IA, HY, and IMP) that may be relevant to the broad disorder (in this case ADHD). Below the subfactor level are symptom components and maladaptive traits, which are then followed by signs and symptoms (6). At this point in its development, there is little information in terms of signs and symptoms for the various syndromes and disorders. Overall, therefore, researchers interested in the HiTOP are not necessarily seeking to classify broad disorder composites but specific dimensions that may have relevance to the broad disorder composites. An individual's psychopathology is conceptualized along the relevant dimensions with varying degrees of severity, and not in terms of distinct categories (6). Since its proposal, emerging empirical data is providing increasing support for the HiTOP approach.

Within HiTOP, ADHD is listed in the antisocial subfactor (which is a blend of the disinhibited externalizing and antagonistic externalizing spectra). Other disorders in this subfactor include antisocial personality disorder, oppositional defiant disorder, conduct disorder, and intermittent explosive disorder. The maladaptive traits for these disorders (primarily related to the disinhibited externalizing spectra) are problematic impulsivity, irresponsibility, theft, distractibility, risk taking, low rigid perfectionism, low ruminative deliberation, and low conscientiousness (6). As problematic impulsivity and distractibility can be seen as corresponding to DSM and ICD ADHD symptom groups for IA and IMP, respectively, it can be extrapolated at this stage that HiTOP defines ADHD only in terms of IA and IMP symptom groups, with the HY not included. Indeed, the motor overactivity that corresponds to the HY symptoms is completely absent in the HiTOP model. Also, as the current HiTOP model does not specify the signs and symptoms for problematic impulsivity and distractibility, the specific symptoms proposed for ADHD IA and ADHD IM in the HiTOP model remain underexplored. Notwithstanding this, ADHD is conceived as an impulsivity disorder with inattention, instead of inattention, hyperactivity, and impulsivity. Overall, there are major differences across HiTOP, DSM, and ICD conceptualizations of the latent structure of ADHD (Supplementary Table 1).

Since the introduction of the DSM-IV, numerous studies have examined the factor structure of ADHD symptoms using different measurement models. The vast majority of earlier studies have used the independent cluster confirmatory factor analysis (ICM-CFA) model and, to a lesser degree, the bi-factor CFA model. More recently, researchers have begun to use more advance approaches, in particular, exploratory structural equation modeling with targeted rotation (ESEM) and S-1 bi-factor CFA modeling. In the S-1 model, the items in one of the group factors are selected (based generally on theory) as reference indicators for the g-factor: that is, the selected group of reference items load only on the g-factor and do not have their own specific factor. So far, no study has evaluated ADHD symptom structure—simultaneously in the same sample—against these seemingly irreconcilable structural constructs, as proposed by DSM, ICD, and HiTOP frameworks. This study aimed to fill this gap. To this end, we will first review and appraise the measurement models in the ADHD literature, in particular their strengths, weaknesses, and limitations, in order to select the best set of candidate models to probe the optimum structure.

The ICM-CFA model is an a priori oblique model in which items load only on their designated factors, i.e., no cross-loadings. Thus, each factor captures the shared variances of its designated items (8). Corresponding to DSM symptom grouping, past studies (involving children and adolescents) have supported ADHD models with separate factors for IA and HY/IM (9–11). The findings have also found support for three-factor models, reflecting both DSM-5 and ICD-10 symptom configurations. However, most researchers have argued in favor of the two-factor model as there was little difference in global fit between the two- and three-factor models, and the two-factor model was more parsimonious (9, 12, 13); moreover, the derived correlations between the HY and IM factors (generally >0.80) were high and deemed lacking adequate discriminant validity between these factors.

Over the last 10 years, studies have increasingly used the bi-factor CFA models to examine the structure of the ADHD symptoms. In general, the bi-factor CFA ADHD model (14, 15) comprises one general ADHD factor (g-factor) and either two (IA and HY/IM) or three (DSM-5-based or ICD-10-based) specific factors. In this model, all the ADHD symptoms load on the g-factor, and the symptoms for each group factor (e.g., IA symptoms) load only onto their own specific factor. The g-factor and specific factors are uncorrelated. As such, the g-factor captures the common variances of all items in the measure, whereas each specific factor captures the unique variances for its own set of symptoms unaccounted by the g-factor. Thus, the specific factors are conceptually and statistically different from “primary factors” in the first-order factor model.

In general, the bi-factor CFA model has demonstrated better fit for the ADHD symptoms than first-order CFA models (see 16). Additionally, studies involving adults have shown better fit for models with three specific factors (IA, HY, IM; or IA, motoric-HY, verbal HY/IM) than with two specific factors (IA and HY/IM; 5, 17, 18). Also for adults, the three-factor model corresponding to ICD-10 configuration has shown better fit than DSM-5 configuration (5, 16, 17).

The IC-CFA and the bi-factor CFA are not without serious limitations. Constraining cross-loadings to zero in ICM-CFA models has been considered excessively restrictive as items in reality are rarely pure indicators of their latent factors, and therefore some degree of construct-relevant association with non-target but conceptually related factors is expected (18). Thus, as pointed out by Marsh et al. (19), the ICM-CFA approach does not generally express the reality of the data set, and yields artifacts of false poor fit. This shortcoming is particularly relevant for the ADHD symptoms given that exploratory factor analysis (EFA) studies have consistently demonstrated cross-loadings for the ADHD symptoms [e.g., (20–22)].

Regarding the bi-factor CFA approach, it has been suggested that such models are prone to yield statistically better-accommodated but non-sense response patterns in the data (8). As such, they will tend to yield a misleadingly better statistical fit than the corresponding first-order factor model, even when this is not actually the case; therefore, the superior fit noted for symmetrical bi-factor ADHD models may reflect a methodological artifact. Moreover, bi-factor CFA models often yield inadmissible solutions, with suboptimal parameters, such as low or even negative loadings of symptoms on designated factors. According to Burns et al. (14) and others (15), the anomalies (i.e., poorly defined factors with poor reliabilities and validities) in symmetrical bi-factor CFA can be explained in terms of improper parameterization of such a model. The bi-factor CFA model assumes that all group factors in the model are interchangeable. That is, they contribute equally toward the g-factor (thus referred as “symmetrical”). However, the findings in virtually all previous bi-factor CFA studies on ADHD have shown that this assumption does not hold (14), as the g-factor is disproportionally loaded with more variances from the HY/IM group of symptoms. To overcome the aforementioned shortcomings of these approaches, two modeling techniques have recently been proposed.

First, Asparouhov and Muthén (23) have developed the exploratory structure equation model (ESEM) with target rotation to overcome the limitations of the ICM-CFA approach. ESEM allows testing of an a priori defined structure (like CFA) while allowing non-zero cross-loadings (like EFA). This approach therefore overcomes a limitation of CFA while retaining its advantages (being model based). As shown in Supplementary Figure 1 (Models 3 and 4), symptoms load on their own designated factors as well as non-designated factors at values close to (but not forced) zero. Indeed, outside of the ADHD field, studies have demonstrated that ESEM is superior to both EFA and CFA approaches for testing factor structures (19, 24).

Second, to overcome interchangeability problems in the bi-factor CFA models, Burns et al. (14), and Eid et al. (15) have introduced the bi-factor CFA S-1 model (also referred as “asymmetrical”). As mentioned earlier, in this model, the items in one of the group factors are selected (based generally on theory) as reference indicators for the g-factor: that is, the selected group of reference items load only on the g-factor and do not have their own specific factor. Other specific factors in the model (that are allowed to correlate with each other) are regressed on the g-factor. The resultant residual variances (i.e., true scores in the group factors modeled as specific factors that are not shared with the g-factor) are inferred as the variances for the specific factors. A feature of the g-factor and specific factors in a bi-factor S-1 model is that they have clear a priori definition and therefore allow for a clear interpretation of findings including their relationships with external correlates.

In addition, structural models also require scrutiny of reliability and external validities. It is necessary to show that the g-factor and specific factors are also clearly defined in the patterns of factor loadings and omega coefficients. Furthermore, the derived factors have to be validated against external measures. In other words, the factors need to demonstrate acceptable reliabilities and external validities (25). For ADHD, existing evidence from bi-factor CFA models shows that although the g-factor is generally clearly defined with acceptable reliability and validity (8, 26, 27), the specific factors (especially HY/IM) are often poorly defined (low or non-significant and sometimes negative loadings) and lack acceptable reliabilities (8, 26, 27).

Given the superiority of the ESEM and S-1 bi-factor approaches, we postulate that these approaches could be better candidates in identifying the optimum factor structure of the ADHD symptoms.

The ESEM approach has been applied in two studies involving ADHD symptoms in children. Arias et al. (28) obtained teacher ratings of preschool children and found stronger support for ESEM models, compared to the corresponding CFA models. The best-fitting model was the bi-factor ESEM model with three specific factors (IA, HY, and IM); notably, while the correlation between the HY and IM factors was 0.807 in the CFA model, it fell to 0.541 when examined using ESEM, thereby indicating support that there is indeed discriminant validity between HY and IM factors in three factor ADHD ESEM models. Rodenacker et al. (27) compared bi-factor CFA and bi-factor ESEM models with two specific factors (IA and HY/IM), three specific factors (IA, HY, and IM), and an incomplete model with one general ADHD and only two specific factors (IA and IM) for parent and teacher ratings of clinically referred children aged 6–18 years (60.4% with primary or secondary ADHD diagnosis). ICD-10-based models were not tested in the study. For both parent and teacher ratings, all models showed good and equivalent model fit, although the specific factors in all models for both respondent types were weakly defined. Recently, in a study involving an adult community sample, Gomez and Stavropoulos (17) found most support for the ESEM model with ICD-10 group factors for IA, motoric HY/IM, and verbal HY/IM. Thus, published studies to date provided evidence that ESEM models may offer more valid and meaningful representations of the latent structure of ADHD symptoms, in line with our postulation.

The bi-factor S−1 CFA approach has been applied to two recent studies that examined the DSM-5-based factor structure of ADHD symptoms (but together with ODD symptoms) in children. Based on trait impulsivity theory, Burns et al. (14) used the HY/IM symptoms as the reference indicators for the g-factor. The trait impulsivity theory posits that ADHD comprises dysfunction in the mesolimbic reward pathway (29), resulting in the development of HY/IM symptoms, and IA symptoms develop later as secondary symptoms, or as expression of distinct mesocortical anomalies. Burns et al. (14) applied both the bi-factor model and the bi-factor S−1 CFA model to ADHD and ODD symptom ratings of children by mothers, fathers, and teachers. The findings from the symmetrical bi-factor CFA models were unsatisfactory, showing (i) anomalous factor loadings, (ii) a weakly defined HY/IM specific factor, and (iii) poor external validities in the associations of the g-factor and specific factors with external correlates (social impairment, academic impairment, and peer rejection). In contrast, the asymmetrical bi-factor S−1 CFA model showed clearly more interpretable results, with (i) well-defined specific factors; (ii) interpretable configuration of HY/IM items loading onto the g-factor; and (iii) expected associations for the g-factor and specific factors with the external correlates.

Junghänel et al. (30) also evaluated the merits of S-1 bi-factor CFA and examined a group of clinic-referred children with ADHD and ODD symptoms and similarly examined symmetrical CFA and bi-factor CFA models, and a series of the S-1 model with (i) HY/IM (based on DSM-5) as the reference factor; (ii) HY/IM (based on ICD-10 grouping) as the reference factor; (iii) HY (based on ICD-10) as the reference factor; and (iv) IM (based on ICD-10) as the reference factor. Their findings indicated that the S-1 models showed better fit than other models. Also, the models with either HY or IM as a reference factor had slightly better fit than the model with HY/IM as the reference factor. In these S−1 models, the g-factor and the IA-specific factors were clearly defined and demonstrated good reliabilities. Thus, these two studies provided preliminary evidence that bi-factor S-1 models offer a more valid and meaningful approach for testing the latent structure of ADHD symptoms.

**Figure 1:**
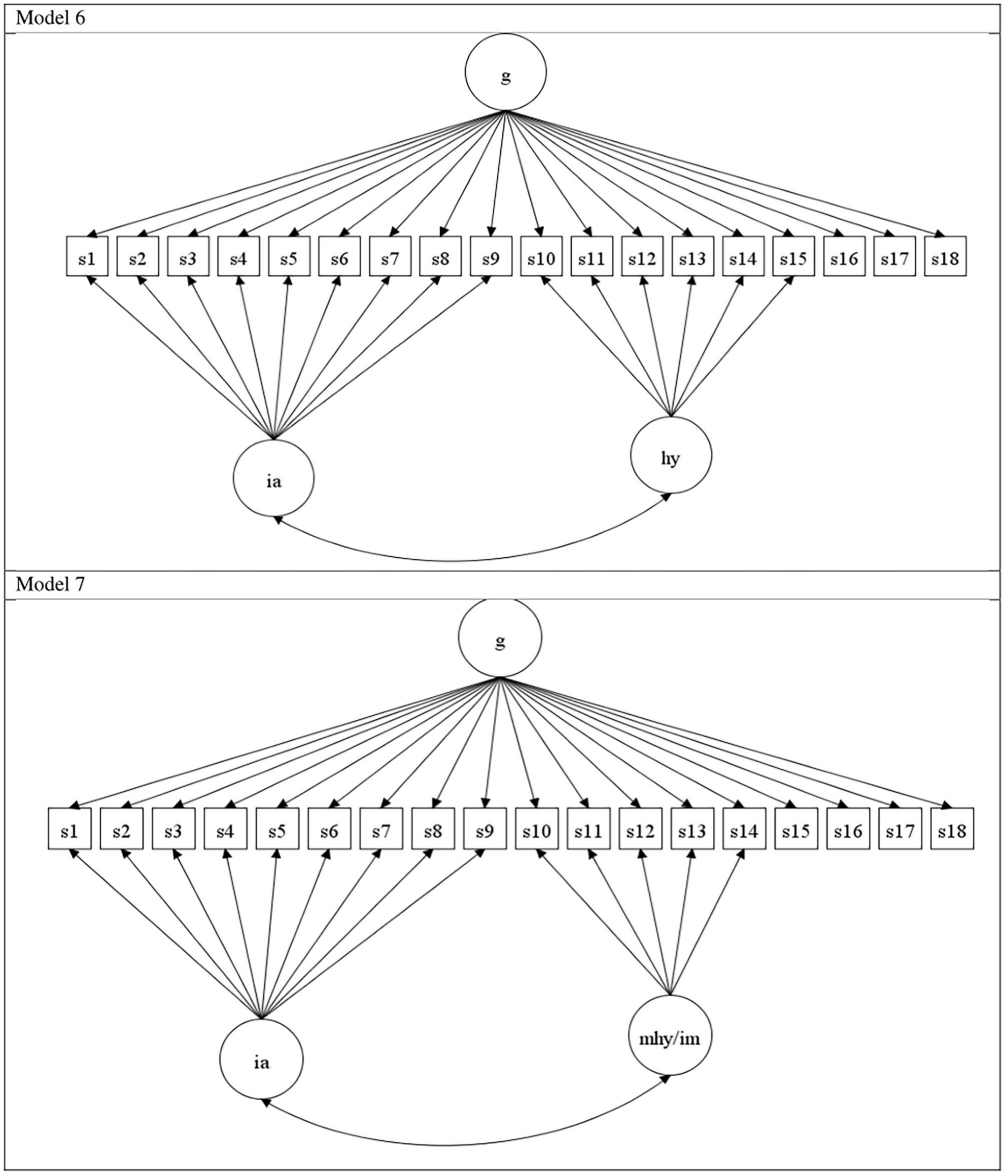
Model 6: s – 1 bit factor, with IM as reference indicators g-factor: & IA and HY as specific factors. Model 7: s – 1 bit factor, with VHY/IM as reference indicators g-factor: & IA and MHY/IMP as specific factors. ia, inattention symptoms; hy, hyperactivity; im, impulsivity; g, general factor ADHD; mhi, motor hyperactivity/impulsivity; vhi, verbal hyperactivity/impulsivity. S1 to s18 refers to the eighteen ADHD symptoms, in the order listed in DSM-IV and Supplementary Table 1.

To date, no study has applied the range of aforementioned models (CFA, ESEM, and S-1 bi-factor CFA) concurrently to evaluate the ADHD symptom structure in a community juvenile sample and also evaluate the differential merits of DSM-5, ICD-10, and HiTOP formulations. To address this gap, the current study sought to examine and compare the structure of ADHD symptoms in children from the general community, using CFA, ESEM, and bi-factor S-1 models—in the context of DSM-5, ICD-10, and HiTOP symptom groupings. Also, as the bi-factor CFA model violates the assumption that all group factors in the model are interchangeable, such models were not tested and reported here as they were deemed digressive and statistically inappropriate for evaluating the factor structure of the ADHD symptoms (but their findings are available upon request). In total, seven ADHD models were compared. These models are described in Figure 1, Supplementary Table 2 and depicted diagrammatically in Supplementary Figure 1. The models tested were:

- CFA two-factor, group factors for IA & HY/IM (Model 1);
- CFA three-factor, group factors for IA, MHY/IM & VHY/IM (Model 2);
- ESEM two-factor, group factors for IA & HY/IM (Model 3);
- ESEM three-factor, group factors for IA, MHY/IM & VHY/IM (Model 4);
- s−1 BCFA, HY/IM reference factor, IA specific factor (Model 5);
- s−1 BCFA, IM reference factor, IA & HY specific factors (Model 6);
- s−1 BCFA, VHY/IM reference factor, IA & MHY/IM specific factors (Model 7).

Models 1, 3, 5, and 6 are DSM-5 based as the different symptom groups correspond to those in DSM-5, whereas Models 2, 4, and 7 are ICD-10 based as the different symptom groups correspond to those in ICD-10. In Model 7, concurrent support for the g-factor and IA-specific factors and lack of support for the MHY/IM would indicate support for the HiTop models as such a model will indicate a model with only IM (since the g-factor is index by IM symptoms) and IA symptoms (since there is a IA specific factor). All the models were tested separately for parent and teacher ratings. Additionally, we probed the reliabilities and external validities of the factors yielded in the models that were deemed potentially good.

## Methods

### Participants

In total, 792 parents and 396 teachers from Victoria, Australia, completed ratings for children from of a community sample. Most parents resided in metropolitan Melbourne (58%) while the remainder (42%) was from regional and rural Victoria. Parents provided ratings (*N* = 792) for 387 (48.9%) girls and 405 (51.1%) boys from 16 randomly selected schools, and teachers rated (*N* = 396) the same 190 (48%) girls and 206 (52%) boys. Thus, 50% of children with parent ratings did not have teacher ratings. The difference in the numbers of parent and teacher ratings was because for many children, teachers did not complete the ratings, despite consent having been granted by parents. The ages of students ranged from 6 to 12 years for both parent (mean = 8.88, SD = 1.68) and teacher (mean = 8.38 years; SD = 1.74) ratings. No significant difference in age between gender was detected for students that rated their parents [*t*_(790)_ = 1.166, *p* = 0.244] and teachers [*t*_(394)_ = 0.122, *p* = 0.903).

Supplementary Table 3 shows the descriptive statistics of the 18 ADHD symptoms for parents and teacher ratings on the Disruptive Behavior Rating Scale (DBRS). Supplementary Table 4 shows the descriptive statistics (mean and SD scores) for the five Strengths and Difficulties Questionnaire (SDQ) scales.

### Measures

#### The Disruptive Behavior Rating Scale—Parent and Teacher Versions [DBRS; (31)]

The DBRS comprises all DSM-IV symptoms for ADHD, Oppositional Defiant Disorder, and Conduct Disorder. For both versions, only the 18 ADHD symptoms (9 IA and 9 HY/IM) were used in the current study. Each symptom is rated on a four-point scale from 0 (never or rarely) to 3 (very often) in terms of occurrence over the previous 6 months.

#### Strength and Difficulties Questionnaire—Parent and Teacher Versions [SDQ; (32)]

The SDQ contains 25 items (categorized into five subscales of five items each: hyperactivity/inattention (HI), emotional symptoms (ES), conduct problems (CP), peer problems (PP), and prosocial behavior (PS). We focused on the ADHD symptoms in the DBRS and not the HI items in the SDQ in the factor analysis as the latter does not provide a complete list of the 18 DSM symptoms. The SDQ items are rated on a three-point scale from 0 (not true) to 2 (certainly true). The five SDQ subscales were used as covariates to test validity for the ADHD factors.

### Procedure

A community sample of parents and teachers of children (between 6 and 12 years of age) were recruited from schools in Victoria, Australia. The study was approved by the University of Ballarat Human Research Ethics Committee, the Victorian Education Department, Catholic Education Office of Victoria, and the principals of participating schools. Following all the ethics and other approvals, classroom teachers from the randomly selected schools were given sealed envelopes containing a letter providing background to the study, the parent version of the DBRS and SDQ, a consent form, a form for parental approval for their children's class teachers to rate their children on the teacher version of the DBRS and SDQ, and a return envelope. These were forwarded to parents through their children. Approximately 1,500 envelopes were distributed. Of these, 792 were returned by parents. The DBRS was completed mostly by mothers (96%). Of the 792 students who had parental approval for their teachers to complete the DBRS and SDQ, 396 teacher questionnaires were also completed.

### Statistical Analysis

Regarding statistical power, the sample size (for parent and for teacher ratings) in the current study is well above the level generally recommended for the factor analyses involving 18 indicator items (i.e., a minimum sample size of 20 × 18 = 360) [see (33)].

All statistical analyses were conducted using Mplus Version 7 (34). As the scores for the ADHD ratings were ordered-categorical scores, we used WLSMV extraction (35). All ESEM models in the study were conducted using geomin (oblique) rotation. For the bi-factor S−1 CFA models, the technique described by Burns et al. (14) was used. For Model 5, we used the HY/IM symptoms as indicators of the g-factor. For Models 6 and 7, we used the motoric HY/IM and verbal HY/IM symptoms, respectively, as indicators of the g-factor. In each model, the specific factors were correlated with each other and regressed on the g-factor.

Given the constraints and issues with CFA mentioned earlier, it can be anticipated that ESEM/EFA models would fit better than corresponding CFA models, and three-factor models, with one extra factor, would fit better than two-factor models. Thus, to establish the best model, we used a sequential four-step model evaluation based on four criteria that include and go beyond global fit (i) model fit criterion, (ii) clarity criterion, (iii) reliability criterion, and (iv) validity criterion. We coined this standardized approach “stepwise algorithm for model selection” (SAMS) procedure. Step 1 examined and compared the global fit values of all models tested. We selected good-fitting models, regardless of whether they differed from each other. As large samples will inflate χ^2^-values, model fit was evaluated using the root mean squared error of approximation (RMSEA), comparative fit index (CFI), and Tucker–Lewis index (TLI). We deemed a model as a potentially good model if all the approximate fit indices (i.e., RMSRA, CFI, and TLI) indicated good fit. According to Hu and Bentler (36), RMSEA, values <0.06 = good fit, <0.08 = acceptable fit, and > 0.08 to 0.10 = marginal fit. For CFI and TLI, values ≥0.95 = good fit, and ≥0.90 = acceptable fit. Where needed, the difference in the fit of nested models was examined using differences in RMSEA (≥ 0.015) and CFI (≥ 0.010) values (37).

In step 2, all models selected as potentially good models were checked for factor clarity by examining the significance of symptom factor loadings (and cross-loadings in ESEM). Factors with more significant loadings of designated symptoms and fewer loadings of non-designated symptoms were considered to be better defined. As this may leave two or more models equally supported, in steps 3 and 4 we examined the reliabilities and external validities of the factors in these equally supported models. The model with better support for reliabilities and validities of the factors was considered the optimum model.

In step 3, omega (ω) values for the factors were computed (38, 39). Relative to coefficient alpha, the ω provides a model based (and better) measure of the internal consistency of a factor (40). This term ω is used in the context of a first order CFA. In a bi-factor model, the term omega hierarchical (ωh) is used to refer to the internal consistency value for the g-factor, and the term omega-subscale (ωs) is used to refer to the internal consistency values for the specific factors (39). According to Reise et al. (41), ωh and ωs values need to be at least 0.50 with values of at least 0.75 preferred for meaningful interpretation of a scale. However, considering this value too stringent for the specific factors, Smits et al. (42) suggested the following for classifying the ωs values: substantial ≥0.30, moderate.20 to <0.30; and low <0.20.

In step 4, to test the external and differential validities of the ADHD g-factor and specific factors in potentially optimum models, the SDQ subscale scores for HI, CP, ES, PP, and PS were regressed on all model-derived factors. The parent SDQ subscale scores were used for models involving parent ADHD ratings, and teacher-rated SDQ scores for teacher-rated ADHD models. The external validity of the ADHD g-factors and specific factors were inferred from significant positive associations with the SDQ HI scale scores. Differences in the patterns of significant positive associations between with the ADHD g-factors and specific factors with the SDQ scale scores were interpreted as evidence of differential validity of the ADHD g- and s-factors.

## Results

As noted earlier, to establish the best model, we went beyond global fit. We used a sequential four-step model evaluation based (coined SAMS) on four criteria: (i) model fit criterion, (ii) clarity criterion, (iii) reliability criterion, and (iv) validity criterion.

### Parent Ratings

#### Step 1: Examining Global Fit of Models Tested

Table 1 shows the fit values for all seven ADHD models tested, based on parent ratings. Our initial step was to identify models by “good fit criteria.” Only Models 4, 6, and 7 met these criteria (good-fit values for RMSEA, CFI, and TLI). When compared using ΔCFI and ΔRMSEA values, there were no differences in fit between different pairs of these models, as the ΔRMSEA and ΔCFI values did not exceed 0.015 and 0.010, respectively.

**Table 1:** Fit of all the models tested in the study.

	**Fit values**			
**Model (M)** **Parent ratings**	**χ^**2**^ (*df*)**	**CFI**	**TLI**	**RMSEA (90% CI)**
M 1: CFA 2-F (IA, HY/IM)	1034.79 (134)	0.937	0.929	0.092 (0.087–0.097)
M 2: CFA 3-F (IA, MHY/IM, VHY/IM)	766.17 (132)	0.956	0.949	0.078 (0.073–0.083)
M 3: ESEM 2-F (IA, HY/IM)	573.51 (118)	0.968	0.959	0.070 (0.064–0.076)
M 4: ESEM 3-F (IA, MHY/IM, VHY/IM)	355.03 (102)	0.982	0.974	0.056 (0.050–0.062)
M 5: BCFA -1-s-F (G, IA), with HY/IM as reference	880.66 (126)	0.948	0.936	0.087 (0.082–0.092)
M 6: BCFA -2-s-F (G, IA, HY), IM reference	498.52 (119)	0.974	0.966	0.063 (0.058–0.069)
M7: BCFA -2-s-F (G, IA, MHY/IM), VHY/IM reference	478.97 (120)	0.975	0.968	0.061 (0.067–0.067)
**Teacher ratings**				
M 1: CFA 2-F (IA, HY/IM)	609.50 (134)	0.978	0.974	0.095 (0.087–0.102)
M 2: CFA 3-F (IA, MHY/IM, VHY/IM)	465.40 (132)	0.984	0.982	0.080 (0.072–0.088)
M 3: ESEM 2-F (IA, HY/IM)	270.56 (118)	0.993	0.991	0.057 (0.046–0.066)
M 4: ESEM 3-F (IA, MHY/IM, VHY/IM)	163.93 (102)	0.997	0.996	0.039 (0.028–0.050)
M 5: BCFA -1-s-F (G, IA), HY/IM as reference	515.77 (126)	0.982	0.978	0.088 (0.081–0.096)
M 6: BCFA -2-s-F (G, IA, HY), IM reference	243.59 (119)	0.994	0.992	0.051 (0.042–0.061)
M 7: BCFA -2-s-F (G, IA, MHY/IM), VHY/IM reference	240.99 (120)	0.994	0.993	0.050 (0.041–0.060)

*F, factor; IA, inattention; HY/IM, hyperactivity/impulsivity; MHY/IM, motoric hyperactivity/impulsivity; VHY/IM, verbal hyperactivity/impulsivity; CI, confidence interval; CFA, confirmatory factor analysis; ESEM, exploratory structural equation modeling; BCFA, bi-factor confirmatory factor analysis; RMSEA, root mean square error of approximation; CFI, comparative fit index; TLI, Tucker-Lewis Index; s-f, specific factor*.

#### Step 2: Examining Item-Factor Loadings in Models 4, 6, and 7

Table 2 shows the factor loadings for Models 4, 6, and 7. It also presents the number of targeted factor loadings and cross-loadings in these models. As shown in Table 2, only Model 7 (i.e., the ICD-10 bi-factor S−1 CFA model with verbal HY/IM as the reference factor) had all target items loading significantly on their own designated factors, and as this is a bi-factor model, there no cross-loadings. Model 6 (i.e., the DSM-5 bi-factor S−1 CFA model with IM as the reference factor) had one target item (symptom relating to “talk”) with negative but not significant loading, on its designated factor, and again as this is a bi-factor model, there was also no cross-loading. Thus, there was reasonable (but not complete) clarity for this model. For Model 4 (the ICD-10 ESEM model with IA, verbal HY/IM, and motoric HY/IM as factors), all target items loaded significantly on their designated factors, and there were 32 items cross-loading significantly on on-targeted factors. Taken together, these findings indicate that Model 7 was the most clearly defined model, Model 6 also had reasonable clarity, and Model 4 was poorly defined.

**Table 2:** Factor loadings for 4, 6, and 7, based on parent ratings.

	**Model 4**			**Model 6**			**Model 7**		
	**IA**	**MHY/IM**	**VHY/IM**	**G**	**IA**	**HY**	**G**	**IA**	**MHY/IM**
Careless -IA1	0.76^**^	0.08^*^	0.02	0.32^**^	0.70^**^		0.31^**^	0.70^**^	
Inattention -IA2	0.69^**^	0.34^**^	0.15^**^	0.56^**^	0.55^**^		0.56^**^	0.56^**^	
Listen -IA3	0.58^**^	0.29^**^	0.28^**^	0.58^**^	0.41^**^		0.57^**^	0.42^**^	
Instruction -IA4	0.86^**^	0.02	0.18^**^	0.43^**^	0.73^**^		0.42^**^	0.73^**^	
Disorganize -IA5	0.84^**^	0.14^**^	0.09^**^	0.43^**^	0.74^**^		0.43^**^	0.75^**^	
Unmotivated -IA6	0.77^**^	0.20^**^	0.01	0.39^**^	0.69^**^		0.39^**^	0.69^**^	
Lose -IA7	0.70^**^	0.20^**^	0.07	0.41^**^	0.61^**^		0.41^**^	0.61^**^	
Distracted -IA8	0.71^**^	0.40^**^	0.18^**^	0.62^**^	0.56^**^		0.62^**^	0.56^**^	
Forgetful -IA9	0.74^**^	0.24^**^	0.20^**^	0.54^**^	0.60^**^		0.54^**^	0.60^**^	
Fidget -HY1	0.47^**^	0.56^**^	0.20^**^	0.61^**^		0.45^**^	0.61^**^		0.46^**^
Seat -HY2	0.60^**^	0.52^**^	0.13^**^	0.57^**^		0.67^**^	0.56^**^		0.67^**^
Run -HY3	0.48^**^	0.69^**^	0.24^**^	0.72^**^		0.45^**^	0.71^**^		0.45^**^
Quiet -HY4	0.41^**^	0.47^**^	0.40^**^	0.69^**^		0.28^**^	0.69^**^		0.29^**^
Motor -HY5	0.23^**^	0.71^**^	0.30^**^	0.69^**^		0.15^**^	0.69^**^		0.17^*^
Talk -HY6	0.19^**^	0.56^**^	0.49^**^	0.75^**^		**−0.03**	0.74^**^		
Blurt -IM1	0.33^**^	0.38^**^	0.63^**^	0.81^**^			0.81^**^		
Wait -IM2	0.39^**^	0.31^**^	0.77^**^	0.91^**^			0.91^**^		
Interrupt -IM3	0.31^**^	0.34^**^	0.79^**^	0.87^**^			0.87^**^		
Omega—ω_h_				74			0.74		
Omega—ω_s_					0.69	0.17		0.60	0.25
**Number of targeted and non-targeted factor loadings**									
Target items (TI)	9	5	4	18	9	6	18	9	5
Significant TI	9	5	4	18	9	5	18	9	5
Non-TI	9	13	14	0	0	0	0	0	0
Significant non-TI	9	12	11	0	0	0	0	0	0

*G, general factor; IA, inattention; MHY/IM, motoric hyperactivity/impulsivity; VHY/IM, verbal hyperactivity/impulsivity. Boldface values indicate factor loadings in the primary dimension; shaded values indicate significant cross-loadings over 0.30 in absolute value, indexing salience*.

* *p < 0.05*,

** *p < 0.01*.

For Model 4, the correlations between IA and motoric HY/IM, IA and verbal HY/IM, and motoric HY/IM and verbal HY/IM were 0.653, 0.473, and 0.663, respectively. The corresponding correlations in CFA version of this model (Model 2) were 0.806, 0.622, and 0.821, respectively. According to Brown (43), when factor correlations are <0.85, discrimination validity between the factors can be inferred. Thus, it can be taken that for Model 4 there was adequate discrimination between motoric HY/IM and verbal HY/IM. For Model 6, the correlation between IA and HY (reflective of a partial correlation between them, controlling for the g-factor) was 0.695, and for Model 7, the correlation between IA and motoric HY/IM (reflective of a partial correlation between them, controlling for the g-factor) was also 0.695. Thus, support for the discrimination between the two factors in Models 6 and 7 can be inferred. Given the findings in Steps 1 and 2, Model 7 and to a lesser degree Model 6 were retained tentatively as our preferred models. We therefore examined the ωh and ωs and external validities of the factors in both these models.

#### Step 3: Examining Reliabilities of Factors in Models 6 and 7

As shown in Table 2, the g-factor in Model 7 had sufficient reliability (ωh values >0.50) for meaningful interpretation (41). Based on guidelines (42) for classifying the ωs values, for this model, the IA-specific factor was substantial, and the value for the motoric HY/IM-specific factor was moderate. For Model 6, the g-factor also showed sufficient reliability (ωh values >0.50). Although the IA-specific factor was substantial, the value for the HY-specific factor was low. Hence, Model 7 met the reliability criterion for all its factors, whereas Model 6 did not meet this for its HY factor.

#### Step 4: Examining Validities of Factors in Models 6 and 7

Table 3 shows the standardized coefficients (from the regression analysis) for the predictions of all SDQ subscales by the factors in Models 6 and 7. For both models, the g-factor predicted significantly and positively all SDQ subscale scores (HI, CP, ES, PP, and PS), and the IA-specific factor predicted significantly and positively the subscale scores for HI, PP, and PS in model 6, and HI, ES, PP, and PS in model 7. For Model 6, the HY-specific factor did not predict significantly any of the SDQ scales scores, and for model 7, the motoric HY/IM-specific factor predicted significantly and positively the subscale score for HI, but not any of the other SDQ subscale scores. Only Model 7 met all criteria in the SAMS procedure.

**Table 3:** Standardized beta coefficients for the predictions of the SDQ subscale scores by the factors in models 6 and 7, based on parent and teacher ratings.

	**HI**	**CP**	**ES**	**PP**	**PS**
**Parent ratings**					
**Model 6**					
ADHD general factor	0.689^***^	0.497^***^	0.063	0.369^***^	0.369^***^
Inattention	0.318^***^	0.094	0.155	0.308^*^	0.308^*^
Hyperactivity	0.132	0.125	0.177	0.014	0.014
**Model 7**					
ADHD general factor	0.664^***^	0.557^***^	0.241^***^	0.192^***^	0.297^***^
Inattention	0.279^***^	0.127^*^	0.250^***^	0.078	0.224^**^
Motoric hyperactivity/impulsivity	0.277^***^	0.118	0.000	0.157	0.158
**Teacher ratings**					
**Model 6**					
ADHD general factor	0.689^***^	0.497^***^	0.063	0.120^*^	0.369^***^
Inattention	0.318^***^	0.094	0.155	0.207	0.308^*^
Hyperactivity	0.132	0.125	0.177	0.042	0.014
**Model 7**					
ADHD general factor	0.697^***^	0.501^***^	0.073	0.119^*^	0.373^***^
Inattention	0.312^***^	0.094	0.171	0.164	0.292^*^
Motoric hyperactivity/impulsivity	0.124	0.114	0.153	0.100	0.027

*HI, hyperactivity/inattention; ES, emotional symptoms; CP, conduct problems; PP, peer problems; PS, prosocial behavior*.

*** *p < 0.001*;

** *p < 0.01*;

* *p < 0.05*.

### Teacher Ratings

#### Step 1: Examining the Global Fit of Models Tested

Table 1 shows the fit values for all seven ADHD models tested based on teacher ratings. Only Models 4, 6, and 7 meet good global fit criteria (good fit values for RMSEA, CFI, and TLI). There were no differences in fit between these models, as the ΔRMSEA and ΔCFI values between different pairs of these models did not exceed 0.015 and 0.010, respectively. Compared to these models, the CFI values for all the other models were substantially worse.

#### Step 2: Examining the Item-Factor Loadings in Models 4, 6, and 7

Table 4 shows the factor loadings for Models 4, 6, and 7. It also provides a summary of the number of targeted factor loadings and cross-loadings in these models. Like the parent ratings, only Model 7 had all target items loading on their own designated factors, and as this is a bi-factor model, there were also no significant cross-loadings. Model 6 had 1 target item (symptom relating to “talk”) not loading significantly on its own designated factor, and again as this is a bi-factor model, there was no cross-loading. For Model 4, all target items loaded on their designated factors, and there were 27 significant cross-loadings on on-targeted factors. Taken together, these findings indicate that Model 7 was the most clearly defined model, Model 6 can be considered as fairly clearly defined, and Model 4 poorly defined.

**Table 4:** Factor loadings for models 4, 6, and 7, based on teacher ratings.

	**Model 4**			**Model 6**			**Model 7**		
	**IA**	**MHY/IM**	**VHY/IM**	**G**	**IA**	**HY**	**G**	**IA**	**MHY/IM**
Careless -IA1	0.94^**^	−0.09	0.00	0.46^**^	0.75^**^		0.48^**^	0.74^**^	
Inattention -IA2	0.84^**^	−0.01	0.19^**^	0.66^**^	0.68^**^		0.67^**^	0.66^**^	
Listen -IA3	0.74^**^	0.04	0.18^**^	0.63^**^	0.59^**^		0.64^**^	0.58^**^	
Instruction -IA4	0.94^**^	−0.23^**^	0.14^**^	0.53^**^	0.78^**^		0.54^**^	0.77^**^	
Disorganize -IA5	0.97^**^	0.04	−0.12^*^	0.49^**^	0.81^**^		0.50^**^	0.80^**^	
Unmotivated -IA6	0.93^**^	−0.06	0.05	0.53^**^	0.75^**^		0.55^**^	0.74^**^	
Lose -IA7	0.79^**^	0.35^**^	−0.27^**^	0.52^**^	0.74^**^		0.54^**^	0.73^**^	
Distracted -IA8	0.65^**^	0.22^**^	0.18^**^	0.74^**^	0.56^**^		0.75^**^	0.55^**^	
Forgetful -IA9	0.89^**^	0.27^**^	−0.27^**^	0.50^**^	0.80^**^		0.52^**^	0.79^**^	
Fidget -HY1	0.38^**^	0.53^**^	0.13^*^	0.78^**^		0.55^**^	0.79^**^		0.54^**^
Seat -HY2	0.25^**^	0.44^**^	0.31^**^	0.80^**^		0.37^**^	0.81^**^		0.35^**^
Run -HY3	0.30^**^	0.55^**^	0.14^**^	0.77^**^		0.45^**^	0.78^**^		0.43^**^
Quiet -HY4	0.27^**^	0.51^**^	0.21^**^	0.79^**^		0.40^**^	0.79^**^		0.39^**^
Motor -HY5	−0.23^**^	0.68^**^	0.13^*^	0.84^**^		0.12^*^	0.85^**^		0.10^**^
Talk -HY6	−0.01	0.50^**^	0.44^**^	0.86^**^		**0.10**	0.89^**^		
Blurt -IM1	−0.15^**^	0.41^**^	0.69^**^	0.91^**^			0.90^**^		
Wait -IM2	0.09^**^	0.10	0.85^**^	0.97^**^			0.97^**^		
Interrupt -IM3	0.09^**^	0.16^**^	0.77^**^	0.94^**^			0.94^**^		
Omega—ω_h_				0.77			0.78		
Omega—ω_s_					0.61	0.14		0.69	0.16
**Number of targeted and non-targeted factor loadings**									
Target items (TI)	9	5	4	18	9	6	18	9	5
Significant TI	9	5	4	18	9	5	18	9	5
Non-TI	9	13	14	0	0	0	0	0	0
Significant non-TI	8	7	12	0	0	0	0	0	0

*G, general factor; IA, inattention; MHY/IM, motoric hyperactivity/impulsivity; VHY/IM, verbal hyperactivity/impulsivity. Boldface values indicate factor loadings in the primary dimension; shaded values indicate significant cross-loadings over 0.30 in absolute value, indexing salience*.

* *p < 0.05*,

** *p <0.01*.

For Model 4, the correlations between IA and motoric HY/IM, IA and verbal HY/IM, and motoric HY/IM and verbal HY/IM were 0.674, 0.506, and 0.692, respectively. The corresponding correlations in the CFA version of this model (Model 2) were 0.842, 0.660, and 0.853, respectively. Thus, for Model 4 there was adequate discrimination between motoric HY/IM and verbal HY/IM. For Model 6, the correlation between IA and HY (reflective of a partial correlation between them, controlling for the g-factor) was 0.796, and for Model 7, the correlation between IA and motoric HY/IM (reflective of a partial correlation between them, controlling for the g-factor) was 0.786. Thus, there was some support for the discrimination between the two factors in Models 6 and 7. Based on all the findings in Steps 1 and 2, Models 6 and 7 were retained tentatively as our preferred models. We therefore examined the ωh and ωs, and external validities of the factors in both models.

#### Step 3: Examining Reliabilities of Factors in Models 6 and 7

As shown in Table 4, for both Models 6 and 7, the g-factors had sufficient reliability (ωh values >0.50) for meaningful interpretation (41). Based on guidelines (42) for classifying the ωs values, the IA-specific factors in both models were substantial and the values for HY (Model 6) and motoric HY/IM (Model 7) specific factors were low. Hence, Models 6 and 7 met the reliability criterion for the general and IA factors, but not for the HY (Model 6) and motoric HY/IM (Model 7) factors.

#### Step 4: Examining Validities of Factors in Models 6 and 7

Table 3 shows the standardized coefficients (from the regression analysis) for the predictions of all SDQ subscales by the factors in Models 6 and 7. For Models 6 and 7, the g-factors predicted significantly and positively HI, CP, PP, and PS, and there was no significant prediction for ES. For both models, IA predicted SDQ HI and PS. HY (Model 6) or Motoric HY/IM (Model 7) did not predict any of the SDQ scale scores. Taken together, the significant positive associations of the ADHD g-factors and specific factors with the SDQ HI scale scores can be interpreted as supporting the external validity of all the ADHD factors in Models 6 and 7. Also, the differences in the patterns of significant positive associations between the ADHD g-factors and specific factors with the SDQ scale scores can be interpreted as evidence of differential validity of the ADHD g- and s-factors.

## Discussion

The current study aimed to evaluate the optimum latent structure of ADHD symptoms within the ICD, DSM, and HiTOP frameworks, by using CFA, ESEM, and bi-factor S-1 models, applied to parent and teacher ratings. Overall, our findings indicated most support for the S-1 bi-factor model (in parent and teacher) comprised of (i) a g-factor based on ICD-10 Impulsivity symptoms as the reference indicators (ωh at 0.78 for teacher; at 0.74 for parent) and (ii) an inattention-specific factor (ωs at 0.69 for teacher; at 0.60 for parent)—as represented by Model 7 in Figure 1, Tables 2, 4. In both, Model 7, the hyperactivity-specific factor however lacked clarity and reliability (ωs at 0.16 for teacher; at 0.25 for parent).

In our findings, the optimum structure of ADHD therefore embodied only the g-factor and inattention-specific factor. The latent structure from both parents' and teachers' ratings converged. Thus, our findings indicate that ADHD is best viewed as a disorder primarily reflecting impulsivity with a separable inattention (but no hyperactivity) component. In essence, ADHD may better be represented by ADID (attention-deficit impulsivity disorder). This model aligns with the HiTOP proposal for ADHD.

In this study, seven ADHD models in total were tested separately for parent and teacher ratings. Additionally, we probed the reliabilities and external validities of the factors yielded. To establish the best model, we devised a four-step sequential stepwise algorithm for model selection (SAMS) procedure, based on (i) model fit criterion, (ii) clarity criterion, (iii) reliability criterion, and (iv) validity criterion.

Supplementary Table 5 shows summaries of the criteria used for selecting the optimum model for both parent and teacher ratings, based on SAMS. For parent ratings, Model 6 (i.e., the ICD-10-based S−1 bi-factor model with motoric HY/IM as the reference factor, and IA and verbal HY/IM as specific factors) and Model 7 (i.e., the ICD-10-based S−1 bi-factor model with verbal HY/IM as the reference factor, and IA and motoric HY/IM as specific factors) were comparable in terms of meeting model fit and validity criteria. In terms of clarity criterion, Model 6 had one item that did not load on its designated factor, whereas for Model 7, all items loaded on their designated factors. In terms of reliability criterion, the g-factor and the IA specific factor in Model 6, but not the verbal HY/IM factor, showed adequate reliabilities. For Model 7, all factors showed acceptable reliabilities. For teacher ratings, Models 6 and 7 were comparable in terms of meeting model fit, reliability, and validity criteria. In terms of clarity criterion, Model 6 had one item that did not load on its designated factor, whereas for Model 7, all items loaded on their designated factors. Given these findings, we adopted Model 7 as our preferred model for both parent and teacher ratings. Our conclusion is consistent with existing literature that have also reported the strongest support for the bi-factor S−1 CFA model with verbal HY/IM as the reference factor (see 30).

Our findings have a number of implications worthy of note. First, in an S−1 model (14, 15), the g-factor has a clear a priori definition. Notably, the reference factor for the preferred S−1 model was verbal HY/IM, which is the impulsivity symptoms as listed in ICD-10, and the g-factor can therefore be best considered as predominantly reflecting impulsivity as formulated by this ICD-10 grouping. This raises the possibility that, overall, ADHD (which corresponds to the g-factor) is best viewed as a disorder reflecting impulsivity. Moreover, in the S−1 model, the variances in the specific factors are residual variances not accounted for by the general actor, and thus, the support for the IA-specific factor can be interpreted as the presence of a separate distinctive psychopathological process represented by predominantly inattention problems. Using the same line of reasoning, the lack of support for motoric HY/IM can be interpreted as the absence of a distinctive disorder reflecting predominantly hyperactive or motor-overactivity problems—above and beyond that captured by the g-factor. Our findings therefore suggest a markedly novel reconceptualization of ADHD, that ADHD is best viewed as a disorder primarily reflecting the latent trait impulsivity characterized by verbal HY/IM, but in addition, there is a separable component of predominantly inattention problems. In essence, ADHD may be re-conceptualized as “ADID” (attention-deficit impulsivity disorder).

Second, the latent structure detected as our preferred ADHD model also provides support to the HiTOP conceptualization of ADHD within the disinhibited externalizing spectrum. This spectrum is characterized by impulsivity (i.e., acting spontaneously on the spur of the moment without consideration for consequences), irresponsibility (i.e., failing to fulfill obligations or act in a dependable manner), distractibility (i.e., inattentive and not completing tasks), risk taking (i.e., sensation-seeking, engaging in potentially dangerous activities in a reckless manner), and (low) perfectionism (i.e., not completing work to acceptable standards). Notably, hyperactivity is a peripheral expression rather than a core driver of psychopathology within this conceptualization (6, 44). Our findings therefore provide preliminary evidence to support the symptom components and maladaptive traits organized by spectrum as proposed by HiTOP. These interpretations were further supported by our findings that in our preferred model (Model 7) for both parent and teacher ratings, motoric HY/IM did not predict any of the SDQ scale scores, including SDQ HI.

Third, as our preferred model (Model 7) had ICD-10-based verbal HY/IM symptoms as the reference indicators for the g-factor, and IA and motoric HY/IM as specific factors, our findings support the ICD-10 grouping of HY/IM symptoms, and the separation of IA, HY, and IM into separate groups. Related to this, in Model 6, for both parent and teacher ratings, the item referring to “talk” did not load significantly on its IM factor. However, this item loaded significantly on its designated verbal HY/IM factor in Model 7. As Model 11 aligns with how the HY/IM symptoms are grouped in ICD-10, this adds further support that ICD-10 grouping of the ADHD symptoms is more appropriate than the DSM-5 groupings of these symptoms.

Fourth, as the HY and IM factors showed high correlations in the three-factor model, there has been a tendency in past studies to favor the two-factor model over the three-factor CFA model for parsimony despite evidence of better fit for the three-factor CFA model. We have argued earlier that the high correlation between the HY and IM may have been artificially inflated due to how CFA models are parameterized. More specifically, because cross-loadings are not modeled in a CFA, the shared variances for items in different symptom groups are diverted toward the factor correlations (45). In support of this, for both parent and teacher ratings, we found higher correlations in CFA models than in corresponding the ESEM models. Indeed, the moderate correlations between the HY and IM factors, and verbal HY/IM and motoric HY/IM factors in the ESEM model, can be interpreted as sufficient support for the separation of the HY- and IM-related dimensions (43), and therefore testing three-factor ADHD models.

Fifth, for the preferred model, across parent and teacher ratings, the g-factor was associated positively with all SDQ subscale scores (HI, CP, ES, PP, and PS), and the IA-specific factor was associated positively with subscale scores for HI and PS (for parents). These findings can be interpreted to mean that other external and internalizing disorders are comorbid with ADHD via their associations with the ADHD impulsivity symptoms (as the g-factor was index by the impulsivity symptoms).

Sixth, although there is an emerging trend to examine the structure of the ADHD symptoms using ESEM with target rotation approach (17, 27, 28), our results provide stronger support for using bi-factor S−1 models, over ESEM models, in research aimed at examining the factor structure of the ADHD symptoms. In our findings, the model with verbal HY/IM symptoms as the reference indicators provided better fit than those of other reference indicators (i.e., with HY/IM, HY, IM, or motoric HY/IM); this means that verbal HY/IM symptoms are likely a more preferable reference factor and should be included in future studies for replication and exploration of bi-factor S-1 ADHD models.

## Limitations

Despite the novelty of our findings, the study has several limitations. The factor structure of ADHD symptoms was examined using DBRS which is DSM-IV based. The reported high comparability in parental information obtained via rating scales and interviews (46) raises the possibility that our findings are likely to be applicable to ADHD symptom reports from clinical interviews. As our sample was a community sample, the findings may not be applicable to clinic-referred children. As noted by Junghänel et al. (30), specific factors could embody higher variance in clinic-referred samples than non-clinical samples, as distinct subtypes may be less observable in the latter. Because teachers are likely to rate more than one child, their ratings may lack independence. While this can be addressed using the robust “sandwich-type” MLR estimator option in Mplus (34), ethics approval did not permit for the collection of identification information that would have allowed this to be applied. Additionally, 50% of children with parent ratings did not have teacher ratings because teachers did not complete or return the ratings for these children, despite consent granted by parents. This may have confounded our findings. Further studies exploring the properties of this model in different samples, involving different sources (e.g., mothers, fathers, teachers, and self), and using different methods of data collection (e.g., interviews and rating scales), controlling for the limitations highlighted here, are warranted.

Our analysis did not include the hierarchical modeling approach, and it is possible that certain aspects of ADHD could be indicators of an externalizing dimension while others of a possible separate neurodevelopmental disorders spectrum (47), and future studies could further explore this aspect. Finally, different taxonomy frameworks (e.g., DSM, ICD) were derived from factor analyses of their own field trial samples as their best-fit models. However, our study conducted a head-to-head comparison of these models in the same dataset, so that, in this comparison, the best-fitting model with the greatest clarity, reliability, and validity (based on the SAMS algorithm) could emerge as the best candidate. This approach is analogous to a head-to-head drug trial of three medications, all previously shown to be effective in treating ADHD in separate studies; a head-to-head comparison using the same research sample can empirically demonstrate which of the three medications has the largest treatment effect. Our empirical evaluation by head-to-head comparison can provide evidence to counter inference from hypothetical reasoning or extrapolation from historical findings. Our findings are, however, preliminary and need to be replicated by other studies using other samples.

## Summary

In summary, this is the first study to examine the factor structure of ADHD symptoms in children from the general community for both parent and teacher ratings using CFA, ESEM, and S-1 CFA procedures concurrently, in relation to conceptual differences in DSM-5, ICD, and HiTOP frameworks. The major findings and interpretations made here raise the possibility that the core symptoms for ADHD are impulsivity and inattention—and not hyperactivity. Thus, the optimum latent structure of ADHD is consistent of only two (impulsivity and inattention) and not three separate symptom groups (hyperactivity, impulsivity, inattention), as proposed in both the major clinical classification systems (DSM-5 and ICD-10). Regarding the impulsivity construct, the constituents in this dimension are in line with ICD-10 configuration—and not DSM-5. In essence, ADHD may be re-conceptualized as “ADID” (attention-deficit/impulsivity disorder). Our findings and interpretation therefore offer a different understanding of ADHD, and preliminary evidence for an entirely novel perspective in ADHD taxonomy—one that aligns with HiTOP conceptualization of ADHD.

## Data Availability Statement

The raw data supporting the conclusions of this article will be made available by the authors, without undue reservation.

## Ethics Statement

The studies involving human participants were reviewed and approved by the University of Ballarat Human Research Ethics Committee. Written informed consent to participate in this study was provided by the participants' legal guardian/next of kin.

## Author Contributions

RG and VS organized the database. RG performed the statistical analysis and was assisted by VS. RG, WC, LL, RK, and DP wrote and edited sections of the manuscript. All authors contributed to manuscript revision and read and approved the submitted version.

## Conflict of Interest

The authors declare that the research was conducted in the absence of any commercial or financial relationships that could be construed as a potential conflict of interest.

## References

[1] American Psychiatric Association. Diagnostic and Statistical Manual of Mental Disorders (5th ed.). American Psychiatric Association (2013). 10.1176/appi.books.9780890425596

[2] American Psychiatric Association. Diagnostic and Statistical Manual of Mental Disorders (4th ed.). American Psychiatric Association (1994).

[3] American Psychiatric Association. Diagnostic and Statistical Manual of Mental Disorders (4th ed., text rev.). American Psychiatric Association (2000).

[4] World Health Organization. The ICD-11 Classification of Mental and Behavioral Disorders: Diagnostic Criteria for Research. World Health Organization (2018).

[5] Stanton K Forbes MK Zimmerman M. Distinct dimensions defining the Adult ADHD Self-Report Scale: Implications for assessing inattentive and hyperactive/impulsive symptoms. Psychol Assess. (2018) 30:1549–59. 10.1037/pas0000604 29878817

[6] Kotov R Krueger RF Watson D Achenbach TM Althoff RR Bagby RM. The Hierarchical Taxonomy of Psychopathology (HiTOP): a dimensional alternative to traditional nosologies. J Abnorm Psychol. (2017) 126:454–77. 10.31234/osf.io/zaadn 28333488

[7] Kotovs R Krueger RF Watson D. A paradigm shift in psychiatric classification: the Hierarchical Taxonomy Of Psychopathology (HiTOP). World Psychiatry. (2018) 17:24–5. 10.1002/wps.20478 29352543 PMC5775140

[8] Morin AJS Marsh HW Nagengast B. Exploratory structural equation modeling. In: Hancock GR Mueller RO editors. Structural Equation Modeling: A Second Course. Charlotte, NC: Information Age Publishing (2013), p. 395–436.

[9] Gomez R Harvey J Quick C Scharer I Harris G. DSM-IV AD/HD: Confirmatory factor models, prevalence, and gender and age differences based on parent and teacher ratings of Australian primary school children. J Child Psychol Psychiatry. (1999) 40:265–74. 10.1111/1469-7610.00440 10188709

[10] Gomez R Burns GL Walsh JA Alves de Moura MA. A multitrait-multisource confirmatory factor analytic approach to the construct validity of ADHD rating scale. Psychol Assess. (2003) 15:3–16. 10.1037/1040-3590.15.1.3 12674720

[11] Willcutt EG Nigg JT Pennington BF Solanto MV Rohde LA Tannock R. Validity of DSM-IVattention deficit/hyperactivity disorder symptom dimensions and subtypes. J Abnorm Psychol. (2012) 121:991–1010. 10.1037/a0027347 22612200 PMC3622557

[12] Gomez R Burns GL Walsh JA Hafetz N. A multitrait-multisource confirmatory factor analytic approach to the construct validity of ADHD and ODD rating scales with Malaysian children. J Abnorm Child Psychol. (2005) 33:241–54. 10.1007/s10802-005-1831-1 15839501

[13] Wolraich ML Lambert EW Baumgaertel A Garcia-Tornel S Feurer ID Bickman L. Teachers' screening for attention deficit/hyperactivity disorder: comparing multinational samples on teacher ratings of ADHD. J Abnorm Child Psychol. (2003) 31:445–55. 10.1023/A:1023847719796 12831232

[14] Burns L Geiser C Servera M Becker SP Beauchaine TP. Application of the bifactor S−1 S−1 model to multisource ratings of ADHD/ODD symptoms: an appropriate bifactor model for symptom ratings. J Abnorm Child Psychol. (2019) 48:881–97. 10.1007/s10802-019-00608-4 31834589 PMC8017439

[15] Eid M Geiser C Koch T Heene M. Anomalous results in g-factor models: explanations and alternatives. Psychol Methods. (2017) 22:541–62. 10.1037/met0000083 27732052

[16] Gibbins C Topley ME Flora DB Weiss MD Tannock R. Evidence for a general factor model of ADHD in adults. J Attent Disord. (2012) 16:635–44. 10.1177/1087054711416310 22076604

[17] Gomez R Stavropoulos V. Confirmatory factor analysis and exploratory structural equation modeling of the structure of Attention-Deficit/Hyperactivity Disorder symptoms in adults. Assessment. (2020). 10.1177/1073191120905892. [Epub ahead of print]. 32062977

[18] Morin A Tran A Cacti H. Factorial validity of the ADHD Adult Symptom Rating Scale in a French community sample: results from the ChiP-ARD Study. J Attent Disord. (2016) 20:530–41. 10.1177/1087054713488825 23729493

[19] Marsh HW Muthén B Asparouhov T Lüdtke O Robitzsch A Morin AJS. Exploratory structural equation modeling, integrating CFA and EFA: application to students' evaluations of university teaching. Struct Equat Model. (2009) 16:439–76. 10.1080/10705510903008220

[20] DuPaul GJ Power TJ Anastopoulos AD Reid R. ADHD Rating Scale—IV: Checklists, Norms, Clinical Interpretation. New York, NY: Guilford Press (1998). 10.1037/t00680-000

[21] Rohde LA Barbosa G Polanczyk G Eizirik M Rasmussen ER Neuman RJ. Factor and latent class analysis of DSM-IV ADHD symptoms in a school sample of Brazilian adolescents. J Am Acad Child Adolescent Psychiatry. (2001) 40:711–8. 10.1097/00004583-200106000-00017 11392350

[22] Döpfner M Steinhausen HC Coghill D Dalsgaard S Poole L Ralston SJ. Cross-cultural reliability and validity of ADHD assessed by the ADHD Rating Scale in a pan-European study. Europ Child Adolescent Psychiatry. (2006) 15:I46–55. 10.1007/s00787-006-1007-8 17177016

[23] Asparouhov T Muthén B. Exploratory structural equation modeling. Struct Equat Model. (2009) 16:397–438. 10.1080/10705510903008204

[24] Marsh HW Morin AJ Parker PD Kaur G. Exploratory structural equation modeling: an integration of the best features of exploratory and confirmatory factor analysis. Ann Rev Clin Psychol. (2014) 10:85–110. 10.1146/annurev-clinpsy-032813-153700 24313568

[25] Park JL Silveira MM Elliott M Savalei Johnston VCH. Confirmatory factor analysis of the structure of adult ADHD symptoms. J Psychopathol Behav Assess. (2018) 40:573–85. 10.1007/s10862-018-9698-y

[26] Cacti HM Morin AJS Bouchez J Baylé FJ. Bifactor models support ICD-10 construct of ADHD against DSM-IV, both in children rated by teachers and self-rated in adults. Europ Psychiatry. (2013) 28:1. 10.1016/S0924-9338(13)75917-0

[27] Rodenacker K Hautmann C Görtz-Dorten A Döpfner M. The factor structure of ADHD-Different models, analyses and informants in a bifactor framework. J Psychopathol Behav Assess. (2017) 39:92–2. 10.1007/s10862-016-9565-7

[28] Arias VB Ponce FP Martínez-Molina A Arias B Núñez D. General and specific attention-deficit/hyperactivity disorder factors of children 4 to 6 years of age: an exploratory structural equation modeling approach to assessing symptom multidimensionality. J Abnorm Psychol. (2016) 125:125–37. 10.1037/abn0000115 26726819

[29] Beauchaine TP Hinshaw SP Pang KL. Comorbidity ofattention-deficit/hyperactivity disorder and early-onset conduct disorder: biological, environmental, developmental mechanisms. Clin Psychol. (2010) 17:327–36. 10.1111/j.1468-2850.2010.01224.x

[30] Junghänel M Rodenacke K Dose C Döpfner M. Applying the bifactor S-1 Model to ratings of ADHD/ODD symptoms: a commentary on Burns et al. (2019)and a re-Analysis. J Abnorm Child Psychol. (2019) 48:905–10. 10.1007/s10802-020-00637-4 32236849

[31] Barkley RA Murphy KR. Attention-Deficit Hyperactivity Disorder: A Clinical Workbook (2nd. ed.). New York, NY: Guilford Press (1998).

[32] Goodman R. The strengths and difficulties questionnaire: a research note. J Child Psychol Psychiatry. (1997) 38:581–6. 10.1111/j.1469-7610.1997.tb01545.x 9255702

[33] Myers NG Suyeon A Ahn YS. Sample size and power estimates for a confirmatory factor analytic model in exercise and sport: a Monte Carlo approach. Res Q Exerc Sport. (2011) 82:412–23. 10.1080/02701367.2011.10599773 21957699

[34] Muthen LK Muthen BO. Mplus user's Guide (7th ed.). Los Angeles, CA: Muthen & Muthen (2012).

[35] Rhemtulla M Brosseau-Liard PÉ Savalei V. When can categorical variables be treated as continuous? A comparison of robust continuous and categorical SEM estimation methods under suboptimal conditions. Psychol Methods. (2012) 17:354–73. 10.1037/a0029315 22799625

[36] Hu LT Bentler PM. Cutoff criteria for fit indexes in covariance structure analysis: conventional criteria versus new alternatives. Struct Equat Model. (1999) 6:1–55. 10.1080/10705519909540118

[37] Chen FF. Sensitivity of goodness of fit indexes to lack of measurement invariance. Struct Equat Model. (2007) 14:464–504. 10.1080/10705510701301834

[38] Arias VB Ponce FP Nunez DE. Bifactor models of attention-deficit/hyperactivity disorder (ADHD): an evaluation of three necessary but underused psychometric indexes. Assessment. (2018) 25:885–97. 10.1177/1073191116679260 27872349

[39] Zinbarg RE Revelle W Yovel I Li W. Cronbach's alpha, Revelle's beta, and McDonald's omegah: their relations with each other and two alternative conceptualizations of reliability. Psychometrika. (2005) 70:123–33. 10.1007/s11336-003-0974-7

[40] McDonald RP. Test Theory: A Unified Treatment. Mahwah, NJ: Lawrence Erlbaum Associates (1999).

[41] Reise SP Bonifay WE Haviland MG. Scoring and modeling psychological measures in the presence of multidimensionality. J Personal Assess. (2013) 95:129–40. 10.1080/00223891.2012.725437 23030794

[42] Smits IAM Timmerman ME Barelds PD Meijer RR. the dutch symptom checklist-90-revised: is the use of subscales justified?. Europ J Psychol Assess. (2015) 31:263–71. 10.1027/1015-5759/a000233

[43] Brown TA. Confirmatory Factor Analysis for Applied Research. New York, NY: The Guilford Press (2006).

[44] Krueger RF Hobbs KA Conway CC Dick DM Dretsch MN Eaton NR. Validity and utility of Hierarchical Taxonomy of Psychopathology (HiTOP): II. Externalizing superspectrum. World Psychiatry. (2021). [Epub ahead of print]. 10.1002/wps.20844 PMC8129870 34002506

[45] Schmitt TA Sass DA. Rotation criteria and hypothesis testing for exploratory factor analysis: implications for factor pattern loadings and interfactor correlations. Educ Psychol Measure. (2011) 71:95–113. 10.1177/0013164410387348

[46] Magnusson P Smari J Sigurdardottir D Baldursson G Sigmundsson J Kristjansson. Validity of self-report and informant rating scales of adult ADHD symptoms in comparison with a semistructured diagnostic interview. J Attent Disord. (2006) 9:494–503. 10.1177/1087054705283650 16481666

[47] Michelini G Barch DM Tian Y Watson D Klein DN Kotov R. Delineating and validating higher-order dimensions of psychopathology in the Adolescent Brain Cognitive Development (ABCD) study. Trans Psychiatry. (2019) 9:261. 10.1038/s41398-019-0593-4 PMC6797772 31624235

