# Supplementary material for: Unraveling the Optimum Latent Structure of Attention-Deficit/Hyperactivity Disorder: Evidence Supporting ICD and HiTOP Frameworks
Source: Front Psychiatry. 2021 May 14;12:666326. doi: 10.3389/fpsyt.2021.666326 (PMC8163219; doi:10.3389/fpsyt.2021.666326)
Supplement: Supplementary file 1 [file Data_Sheet_1.docx]

**Supplementary Materials**

**Unravelling the optimum latent structure of Attention-Deficit/Hyperactivity Disorder:
 Evidence Supporting ICD and HiTOP Frameworks**

**Supplementary Table S1**

*Summary of Discrepancies between DSM-5, ICD and HiTOP ADHD Symptom Groupings*

| ADHD symptom groups | DSM-5 | ICD | HiTOP |
| --- | --- | --- | --- |
| Inattention | (IA) 9 items  Careless (IA1) Inattention (IA2) Listen (IA3)  Instruction (IA4); Disorganized (IA5) Unmotivated (IA6) Lose (IA7)  Distracted (IA8) Forgetful (IA9) | (IA) 9 items  Careless (IA1) Inattention (IA2) Listen (IA3) Instruction (IA4) Disorganized (IA5) Unmotivated (IA6) Lose (IA7)  Distracted (IA8) Forgetful (IA9) | IA  Items unspecified |
| Hyperactivity | HY (6 items)  Fidget (HY1)  Seat (HY2)  Run (HY3)  Quiet (HY4)  Motor (HY5)  Talk (HY6) | Motoric HY/IM (5 items)  Fidget (MHY/IMH1)  Seat (MHY/IMH2)  Run (MHY/IMH3)  Quiet (MHY/IMH4) Motor (MHY/IMH5) | (absent) |
| Impulsivity | IM (3 items)  Blurt (IM1)  Wait (IM2)  Interrupt (IM3) | Verbal HY/IM (4 items)  Talk (VHY/IM1)  Blurt (VHY/IM2)  Wait (VHY/IM3)  Interrupt (VHY/IM4) | IM  Items unspecified |

IA= Inattention; HY= Hyperactivity; IM = Impulsivity; H/IM = Hyperactivity/Impulsivity.

**Supplementary Table S2**

*ADHD Models Compared in the Study*

| Model # | Brief Description | Based on |
| --- | --- | --- |
| 1 | CFA two-factor, group factors for IA & HY/IM | DSM-5 - as the IA and HY/IM symptom groups correspond to DSM-5 |
| 2 | CFA three-factor, group factors for IA, MHY/IM & VHY/IM | ICD-10 - as the IA, MHY/IM & VHY/IM symptom groups correspond to ICD-10 |
| 3 | ESEM two-factor, group factors for IA & HY/IM | DSM-5 - as the IA and HY/IM symptom groups correspond to DSM-5 |
| 4 | ESEM three-factor, group factors for IA, MHY/IM & VHY/IM | ICD-10 - as the IA, MHY/IM & VHY/IM symptom groups correspond to ICD-10 |
| 5 | s - 1 BCFA, HY/IM reference factor, IA specific factor | DSM-5 - as the IA and HY/IM factors correspond to DSM-5 |
| 6 | s - 1 BCFA, IM reference factor, IA & HY specific factors | DSM-5 - as the IA, HY & IM symptom groups correspond to DSM-5 |
| 7 | s - 1 BCFA, VHY/IM reference factor, IA & MHY/IM specific factors | ICD-10– as IA, MHY/IM & VHY/IM symptom groups correspond to ICD-10; |

*Note*. IA = inattention; HY/IM =hyperactivity/impulsivity; MHY/IM = motoric hyperactivity/impulsivity; VHY/IM = verbal hyperactivity/impulsivity; CFA = confirmatory factor analysis; ESEM = exploratory structural equation modeling; BCFA = bi-factor confirmatory factor analysis. HiTOP = Hierarchical Taxonomy of Psychopathology.

**Supplementary Table S3**

*Mean (Standard Deviation) Scores for ADHD Symptoms for Parent and Teacher Ratings*

|  | Parent | | Teacher | |
| --- | --- | --- | --- | --- |
| Brief descriptions of symptoms | Mean | Std. Deviation | Mean | Std. Deviation |
| S1 Careless (IA1) | 0.85 | 0.74 | 0.82 | 0.85 |
| S2 Inattention (IA2) | 0.47 | 0.65 | 0.68 | 0.82 |
| S3 Listen (IA3) | 0.76 | 0.76 | 0.36 | 0.63 |
| S4 Instruction (IA4) | 0.78 | 0.79 | 0.59 | 0.79 |
| S5 Disorganized (IA5) | 0.61 | 0.74 | 0.57 | 0.80 |
| S6 Unmotivated (IA6) | 0.71 | 0.86 | 0.60 | 0.86 |
| S7 Lose (IA7) | 0.57 | 0.72 | 0.40 | 0.70 |
| S8 Distracted (IA8) | 1.00 | 0.82 | 0.85 | 0.93 |
| S9 Forgetful (IA9) | 0.67 | 0.75 | 0.45 | 0.73 |
| S10 Fidget (HY1) | 0.61 | 0.87 | 0.42 | 0.77 |
| S11 Seat (HY2) | 0.33 | 0.61 | 0.34 | 0.67 |
| S12 Run (HY3) | 0.42 | 0.73 | 0.27 | 0.59 |
| S13 Quiet (HY4) | 0.35 | 0.69 | 0.30 | 0.62 |
| S14 Motor (HY5) | 0.66 | 0.91 | 0.29 | 0.62 |
| S15 Talk (HY6) | 0.88 | 0.93 | 0.59 | 0.83 |
| S16 Blurt (IM1) | 0.61 | 0.76 | 0.35 | 0.66 |
| S17 Wait (IM2) | 0.51 | 0.71 | 0.35 | 0.67 |
| S18 Interrupt (IM3) | 0.76 | 0.80 | 0.40 | 0.73 |

Range for all symptoms rated by parents and teachers were 0 to 3.

**Supplementary Table S4**

*Mean (Standard Deviation) Scores for ADHD and SDQ Subscales for Parent and Teacher Ratings*

|  | Parent | Teacher |
| --- | --- | --- |
| ADHD - Inattention | 6.40 (5.14) | 5.32 (6.12) |
| ADHD - Hyperactivity/Impulsivity | 5.10 (5.12) | 3.31 (5.01) |
| SDQ - hyperactivity/inattention | 2.82 (2.48) | 2.41 (2.58) |
| SDQ - conduct problems | 1.47 (1.72) | 0.91 (1.55) |
| SDQ - emotional symptoms | 2.09 (2.06) | 1.30 (1.89) |
| SDQ - peer problems | 1.48 (1.64) | 1.40 (1.78) |
| SDQ - prosocial behavior | 1.63 (1.84) | 2.35 (2.40) |

**Supplementary Table S5**

*Summaries of the Criteria Used for Selecting the Optimum Model For both Patent and Teacher Ratings Based on Stepwise Algorithm for Model Selection (SAMS)*

| Criterion | Standard | Model 4 | Model 6 | Model 7 |
| --- | --- | --- | --- | --- |
| Parent Ratings | | | | |
| Model Fit | Good fit | Met | Met | Met |
| Clarity | Factors with all significant designated loadings & no significant cross-loadings | Not met | Mostly met (1 item not on designated factor) | Completely met |
| Reliability | omega (ω_h_) ≥ .50; ω_s_ ≥ .20 | Not tested | Met for g & IA, not for HY | Met for all factors. |
| Validity | Significant positive associations with SDQ scale scores | Not tested | Met for all factors | Met for all factors |
| Teacher Ratings | | | | |
| Model Fit | Good fit | Met | Met | Met |
| Clarity | Factors with all significant designated loadings & no significant cross-loadings | Not met | Mostly met (1 item not on designated factor) | Completely met |
| Reliability | omega (ω_h_) ≥ .50; ω_s_ ≥ .20 | Not tested | Met for g & IA, not for HY | Met for g & IA, not for MHY/IM |
| Validity | Significant positive associations with SDQ scale scores | Not tested | Met for all factors | Met for all factors |

*Note*. IA=Inattention; sf=specific factor.

**Supplementary Figure S1**

*Models Tested in the Study.*

| Model 1: CFA 2-F (group factors = IA, HY/IM) |
| --- |
| 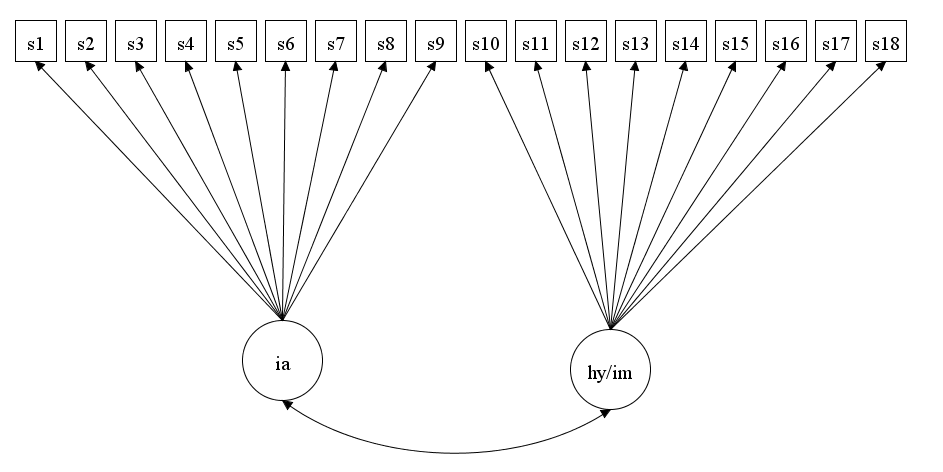 |
| Model 2: CFA 3-F (IA, MHY/IM & VHY/IM) |
| 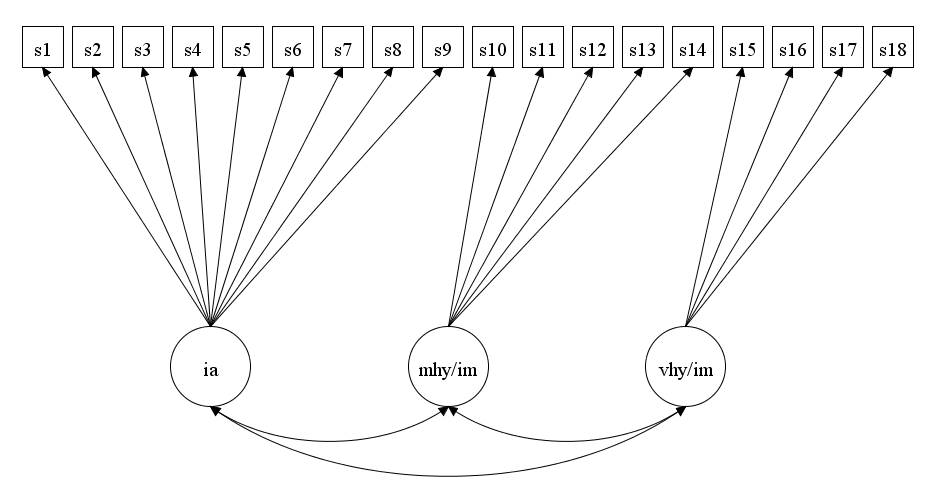 |

| Model 3: ESEM 2-F (IA & HY/IM) |
| --- |
| 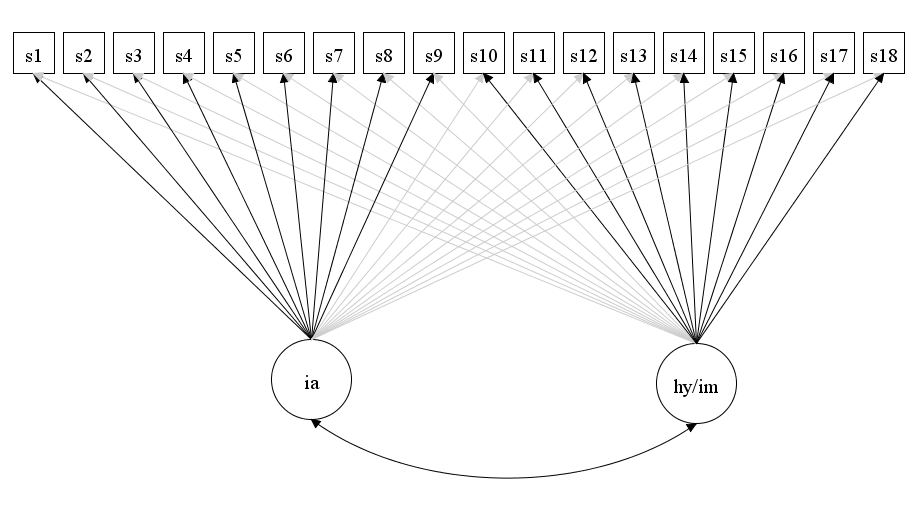 |
| Model 4: ESEM 3-F (IA, MHY/IM & VHY/IM) |
| 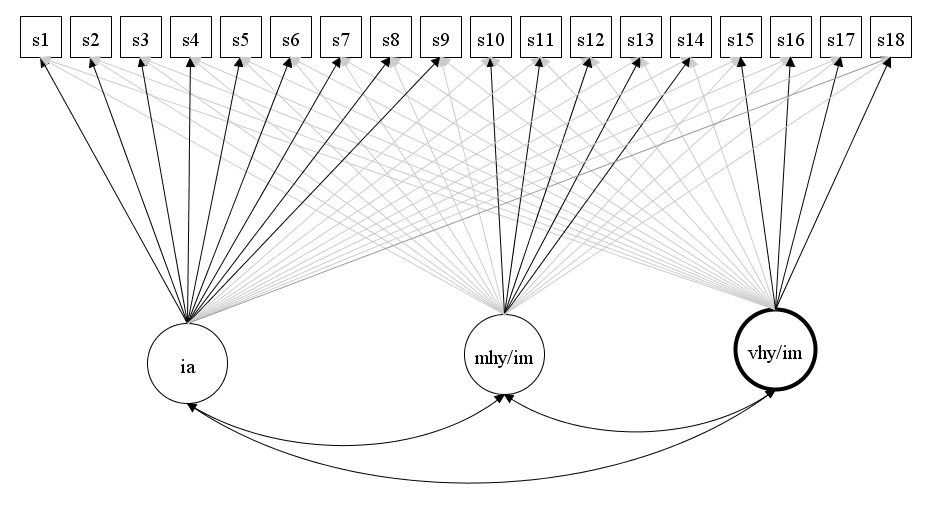 |

| Model 5: s – 1 bi-factor, with HY/IM as reference & IA as specific |
| --- |
| 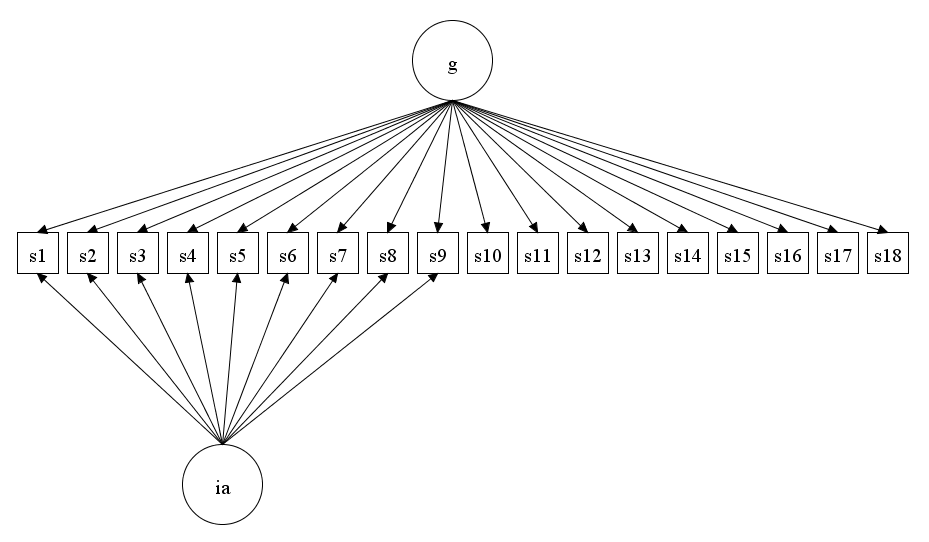 |
| Model 6: s – 1 bi-factor, with IM as reference, & IA and HY as specific |
| 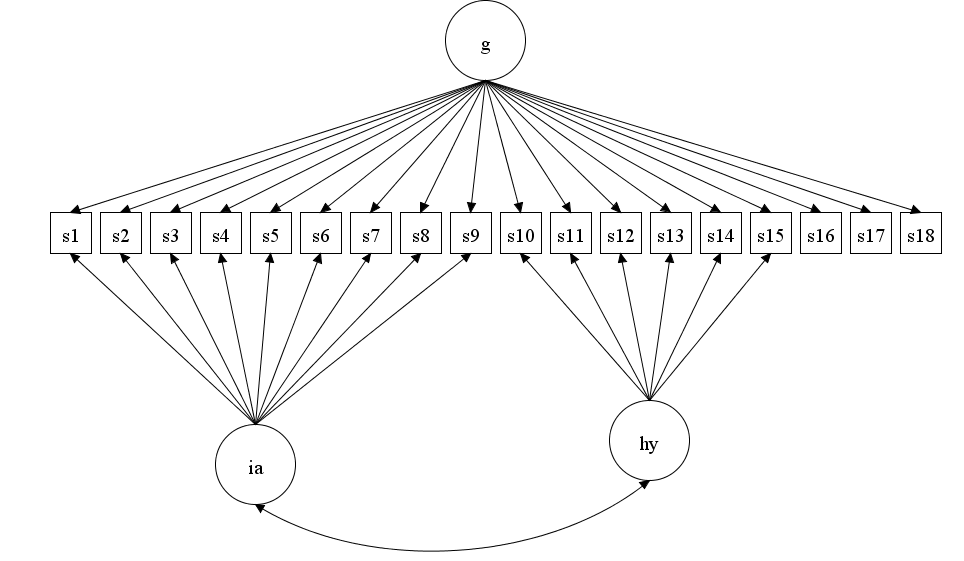 |

| Model 7: s – 1 bi-factor, with VHY/IM as reference, & IA and MHY/IMP as specific |
| --- |
| 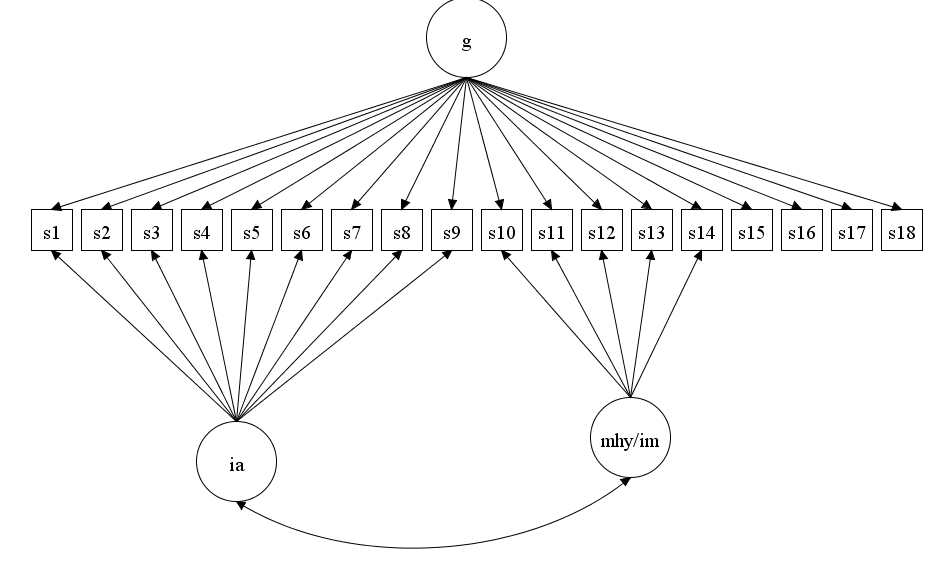 |
| Note. ia = inattention symptoms; hy = hyperactivity; im = impulsivity; g = general factor for ADHD; mhi = motor hyperactivity/impulsivity; vhi = verbal hyperactivity/impulsivity. S1 to s18 refers to the eighteen ADHD symptoms, in the order listed in DSM-IV and table S1. |
